# Health-Related Quality of Life among Rural-Urban Migrants Living in Dhaka Slums: A Cross-Sectional Survey in Bangladesh

**DOI:** 10.3390/ijerph181910507

**Published:** 2021-10-07

**Authors:** Kamrun Nahar Koly, Md. Saiful Islam, Daniel D Reidpath, Jobaida Saba, Sohana Shafique, Md. Razib Chowdhury, Farzana Begum

**Affiliations:** Health System and Population Studies Division, International Centre for Diarrhoeal Disease Research, Bangladesh (icddr,b), Mohakhali, Dhaka 1212, Bangladesh; islam.msaiful@outlook.com (M.S.I.); daniel.reidpath@icddrb.org (D.D.R.); kazi.jobaida.saba@gmail.com (J.S.); sohana.shafique@icddrb.org (S.S.); razib@icddrb.org (M.R.C.); farzanab@icddrb.org (F.B.)

**Keywords:** HRQoL, migrants, slum dwellers, physical health, urban mental health, Bangladesh

## Abstract

Background: The study aimed to assess the health-related quality of life (HRQoL) and its associated factors among urban slum dwellers who migrated from different rural parts of Bangladesh. Methods: The present study analyzed data from a Migration and Mobility Determinants on Health survey and was conducted in 2017 among 935 migrant slum dwellers of Dhaka city (North & South) and Gazipur City Corporations, as a part of the icddr,b’s Urban Health and Demographic Surveillance System (UHDSS). The face-to-face interviews were conducted with the adult population by using a semi-structured questionnaire that included variables related to socio-demographics, migration, occupation, and HRQoL (SF-12). Bivariate and multiple linear regression analyses were performed to determine the factors associated with HRQoL. Results: The mean (±SD) scores of physical component summary (PCS), and mental component summary (MCS) were 57.40 ± 22.73 and 60.77 ± 22.51, respectively. As per multiple regression analysis, lower PCS scores were associated with having older age, being female, and not having any job. Mean MCS scores were significantly lower among participants who reported having older age, not having any job, not working/ less working hours (≤8 h/day), as well as increased work-related stress in the current urban slum. Conclusions: The findings suggest that available urban social protection programs should include a comprehensive social safety net for the improvement of the slum infrastructure as well as proper health care and risk mitigation plans at workplaces.

## 1. Introduction

Globally, urban populations have, in absolute and relative terms, been growing for the past century, and the United Nations (UN) projects that the global urban population will increase by around 1.3 billion by 2030, while the rural population will essentially remain constant [1,2,3]. The global phenomenon is also reflected in Bangladesh, where the percentage of the population living in urban areas has increased from 5% to 27% for 40 years since its independence, and nearly 65 million people currently live in urban areas [4,5]. Almost half the urban population (47.2%) live in slums [6]. As a result of the continued rural tourban migration, the number of informal settlements has accelerated in Bangladesh, with a recent census estimating approximately 14,000 slum settlements across the country [7,8].

A rapid shift from an agrarian to an industrial economy and the concomitant creation of urban occupational opportunities have incentivized many individuals to migrate from rural to urban areas. This migration is not driven by jobs alone, and other contributing factors include natural disasters, economic stagnation, poverty, political and religious violence, and an expectation of a better life [3,9,10,11,12]. However, migrating to a new environment, with a different pattern of living, food consumption, climatic changes, more crowded conditions, and so forth, may increase individuals’ exposures to a number of additional risk factors for different diseases [13]. The urban poor living in slums may be particularly affected, given a disproportionate exposure to poor water, sanitation, and hygiene (WASH) [14], as well as air pollution, all of which are associated with the lower health-related quality of life (HRQoL) [15].

HRQoL is a multidimensional construct that encompasses physical, mental, and social health dimensions [16]. While a large number of studies have looked at migration from developing to developed countries, far less attention has been paid to internal migrants within developing countries, where rural–urban migration has become more significant than international migration [17]. A wide range of global studies has reported lower HRQoL for adults living in urban slums compared to the general population [18,19,20]. The lower HRQoL is associated with various factors, including age, sex, marital status, education, household income, occupation, living environment, working hours, and migration-related stress [21,22,23,24,25].

There is rich global literature on the health of urban slum dwellers. Likewise, in Bangladesh, slightly more than half of all the health studies on urban slum dwellers focused on nutrition, maternal and child health, health care services delivery, and disease-specific incidence and prevalence. Neither in Bangladesh nor elsewhere in the developing world, does one tend to see studies measuring a more encompassing notion of health, such as HRQoL [26,27,28,29,30]. Furthermore, given the known relationship between migration and HRQoL and the fact that slums are urban sinkholes for internal migration, the lack of HRQoL research on migration among urban slum dwellers is a critical knowledge gap. Indeed, overall urban health and well-being cannot be strengthened without considering the HRQoL of internal migrants arriving in urban slums. This study aimed to address the gap by assessing the HRQoL and its associated factors among migrants to urban slums in Dhaka and Gazipur cities, Bangladesh.

## 2. Materials and Methods

### 2.1. Study Design

A cross-sectional survey on the determinants of migration, mobility, and health in residents of urban slums in Dhaka (North & South) and Gazipur City Corporations was conducted in 2017. Data were drawn from an ongoing Urban Health and Demographic Surveillance System (UHDSS), which gathers information on basic demographic characteristics and events including birth, death, marriage, and divorce, migrations (in, out, and internal), household split, fertility regulation, as well as safe motherhood and newborn care practices [9]. The population covered by the UHDSS is about 120,000 people living in 30,000 households.

### 2.2. Study Sites

In Bangladesh, city corporations are the highest level of urban local governments and one of the country’s major urban focal points, with the most advanced administrative and technical capabilities. In the census of slum areas in 2014, a total of 13,938 slums were counted covering all cities and other urban areas of Bangladesh, out of which 33.62% were from Dhaka North (11.80%), Dhaka South (12.59%), and Gazipur (9.23%) City Corporations [9]. Therefore, the current study was conducted among the selected slums of three major city corporations of the Dhaka division: Dhaka North City Corporation (situated in the northern zone with an area of 196.22 km^2^ and an approximate population of 2.5 million), Dhaka South City Corporation (located in the southern site with an area of 109.251 km^2^ and an approximate population of 12 million), and Gazipur City Corporation (situated in the southern zone with an area of 329.23 km^2^ and an approximate population of 2.5 million) [31,32,33,34].

### 2.3. Participants and Selection Procedures

A total of 1100 adult residents aged 18 years of age or more (equally divided between male and female participants) were randomly selected and interviewed from the UHDSS database. Of them, 935 subjects who had migrated to the current slum areas from their rural residence were included in the final analysis.

### 2.4. Study Instruments

Each interview relied on a semi-structured Bangla questionnaire including informed consent with sections on socio-demographic characteristics, migration history, and the SF-12 [35]. The face-to-face interviews were conducted by experienced female surveillance/skilled workers who had at least 12 years of schooling.

#### 2.4.1. Socio-Demographics

Socio-demographic data included age, sex, marital status, and education from the existing database of the UHDSS. As part of the Migration and Mobility Determinants on Health survey, the participants were also asked about migration-related information about when they migrated and the reasons for migration.

#### 2.4.2. Living Environment (Contrasting of the Present Urban and Former Rural Dwelling Status)

The participants were asked to compare their present status of living in urban slums with their former rural places of residence. Comparisons were made between housing environments, toilet facilities, water quality, and open space. They were explicitly asked whether their current residence was “worse”, “the same as before”, or “better” than their previous rural residence.

#### 2.4.3. Occupation-Related Variables

Sources of income and working hours in current residence were asked during the survey. As slum dwellers are engaged in various risky occupations and have no defined working hours, we added two additional self-reported questions that measured the work-related risk and stress in the current place (e.g., high, moderate, and not significant).

#### 2.4.4. Short-Form Health Survey (SF-12)

The validated Bangla version of the SF-12 was used to assess the HRQoL [36,37]. The SF-12 is the abridged version of the 36-item Health Survey (SF-36) and one of the most widely used instruments globally in epidemiological surveys to measure HRQoL [35,38]. The SF-12 is a brief, easy-to-use, psychometrically sound and robust instrument that is validated and translated in a wide range of languages in different countries [39]. The scale has 12 items comprising binary and Likert-scale answers that measure physical and mental health-related quality of life. Of the 12 items, six relate to physical health, five relate to mental health, and one question combines the dimensions of both physical and mental health. The SF-12 has eight subscales: general health, physical functioning, role limitations due to physical health, role limitations due to mental health, mental health, body pain, vitality, and social functioning, which provide a physical component summary (PCS) score and a mental component summary (MCS) score. The PCS and the MCS each range from 0 to 100, with a lower score indicating worse HRQoL and a higher score indicating better HRQoL. The mean score was 50 (SD = 10) for the American general population, so the cutoff value of 50 can be the standard indication of the HRQoL for the general population, since no cut-off score has been found from the developing countries [40].

### 2.5. Statistical Analysis

Two statistical software packages were used to analyze and manage the data (IBM SPSS Statistics version 25 and STATA version 13). Data cleaning, coding, and sorting were performed using SPSS. Linear regression analyses were performed using STATA to model the total scores of PCS and MCS in both unadjusted and adjusted analyses. The bivariate regression analyses were used to determine the candidate-independent variables for inclusion in the multiple regression analyses (*p* < 0.25). Finally, multiple regression analyses were performed individually to model both PCS and MCS involving all candidate-independent variables selected from the bivariate regression analyses. In addition, Pearson product–moment correlations were estimated between the different subscales and domains of SF-12. A *p*-value less than 0.05 was considered significant for all statistical tests.

### 2.6. Ethics

Informed consent was obtained from all participants at the time of data collection. The study was approved by the Institutional Review Board (IRB) of the icddr,b that includes a Research Review Committee and an Ethics Review Committee.

## 3. Results

### 3.1. General Profile of Participants

A total of 935 participants were included in the final analysis. Of them, more than half were females (54.2%), half of them had no formal education (51.0%), and most were married (89.7%) (Table 1). The majority had left their rural residences more than 20 years earlier (41.8%). The participants also reported that they had primarily migrated for family reasons (49.8%) and new job opportunities (42.2%). A sizable majority (61.5%) of the participants were daily wagers at the time of the interview. Only 13.6% of participants worked currently as support service staff (e.g., drivers, doorkeepers, and house caretakers) with negligible salaries. With regard to current working hours, 40.3% of participants reported not working, 30.7% reported working for ≤8 h per day, and 29.0% worked for >8 h per day. Most participants reported high work-related risk (85.9%) and stress (83.5%) while living in their current residence.

When comparing their current living situations with their previous rural residence, 54.3% of participants reported a worse current housing environment, 58% reported a worse current water supply, and 83.4% reported worse housing space (Table 1). In contrast, 51.9% reported better current toilet facilities.

### 3.2. HRQoL

Table 2 presents the descriptive statistics and correlations among SF-12 subscales and its two major dimensions (PCS and MCS). Among the 8 subscales, the highest mean score was found in the social functioning subscale (76.63 ± 29.53); in contrast, the general health perception subscale exhibited the lowest mean scores (36.28 ± 28.71). The mean scores of PCS and MCS were 57.40 (SD = 22.73) and 60.77 (SD = 22.51), respectively. The PCS and MCS scores were significantly and positively correlated with each other (*r* = 0.57; *p* < 0.001).

The bivariate regression analyses (Table 3) identified age, sex, marital status, education level, duration of migration, source of income, reasons for migration, working hours, and work-related risk and stress in the current residence as candidate variables for inclusion in the multiple regression analysis for PCS (*p* < 0.25). After statistical adjustment, participants’ age was negatively and significantly associated with PCS scores (*p* < 0.001); females had significantly lower mean PCS scores than males (*p* < 0.001); and all employment categories had lower mean PCS scores compared with the category “jobs” (*p* =< 0.001–0.009). Being divorced/widowed, having no education, longer duration of migration, and not working/ less working (≤8 h/day), were significantly (*p* < 0.05) associated with lower PCS scores in the bivariate analyses but were not significant in the multiple regression analysis.

The bivariate regression analyses (Table 4) identified age, sex, marital status, education level, duration of migration, duration of stay in the current place, source of current income, reasons for migration, current working hours, and work-related stress in the current place as candidate variables for inclusion in the multiple regression analysis for MCS (*p* < 0.25). Of the candidate variables, after statistical adjustment, participants’ age was negatively and significantly associated with MCS scores (*p* < 0.001); employment category “daily wager and others” exhibited lower mean MCS scores compared with the category “jobs” (*p* = 0.031–0.004); those reporting currently not working or working for ≤ 8 h/day had lower mean MCS scores compared with those who worked for >8 h/days (*p* = 0.001–0.002); and those reporting increased work-related stress in the current residence had lower mean MCS scores than those who did not (*p* = 0.011). Being female, divorced/ widowed, longer duration of migration, and having no education were significantly (*p* < 0.05) associated with lower MCS scores in bivariate analysis, but they were not significant in the multiple regression analysis.

## 4. Discussion

Due to overcrowding, congestion, and lack of proper sanitation and water, underprivileged rural migrants in urban slums may experience poor HRQoL [14,15]. To date, there are no peer-reviewed studies that have investigated HRQoL among the lower-income adult population who migrated from different parts of the country to slum areas of Dhaka city. It is, however, critical to investigate the HRQoL among slum dwellers in order to understand both their physical and mental health. In the present study, the HRQoL was lower among migrant slum dwellers and was significantly associated with migration-related stressors along with work-related experiences in the current place. 

Among the SF-12’s eight subscales, the highest mean score was found in the social functioning; in contrast, the general health perception subscale exhibited the lowest mean scores. The slum dwellers mostly live in a densely populated environment, so they are more likely to live as a close community with a greater rate of social interaction [10,41]. Hence, their social functioning score was high. This is consistent with previous studies of Myanmar migrants in Thailand and young rural–urban migrants in China, which reported that the majority of respondents mentioned their health problems (either physical or emotional) did not limit their social activities most or all the time [42,43]. The participants’ lower score in general health perception is possibly due to lack of healthcare-seeking behaviors, less access to health facilities, exposure to poor physical infrastructure, working in undesirable conditions, or a lack of affordable health care [44,45].

In our study, the mean scores of PCS and MCS were 57.40 (SD = 22.73) and 60.77 (SD = 22.51), respectively. The mean scores of PCS and MCS were lower than a previous study conducted among Iraqi immigrants settled in Malaysia using the SF-36 instrument [46]. In the same country, another study conducted with rural community using the SF-12 instrument reported higher PCS scores and lower MCS scores compared to those in the present study [47]. Another study reported lower PCS and MCS scores among Latin-American immigrants in Span using the SF-36 instrument [48]. However, it is not completely justifiable to compare the previous studies due to the different attributes, including diverse ethnicity, age groups, cultural backgrounds, living situation, infrastructure, health care facilities, validity of the tools, and methodologies used in the previous studies. The lower HRQoL demonstrated in our study should therefore be taken into consideration by the concerned authorities.

In the current study, in terms of both PCS and MCS, the HRQoL was comparatively higher in participants who reported a shorter duration of time since migration (≤5 years), and the score became relatively lower with the longer duration of migration (e.g., gradually in 6–10 years, 11–15 years, 15–20 years, and >20 years). However, the duration of migration was not significant in the multiple regression. Participants with older age were significantly associated with lower PCS and MCS scores. This can possibly be due to the fact that young migrants were more active about finding new opportunities and building a better life for the future [43,49,50]. Elderly people dwelling in slum areas are typically long-term migrants having less ability to perform manual labors, becoming an economic burden on their families, at risk of developing chronic diseases, and unable to take risky jobs. The poor HRQoL among them may be due to loneliness, anxiety, insomnia, etc. These factors, along with the compromised housing structure of slums, make them prone to have a lower HRQoL [10,51,52,53,54,55,56,57,58,59].

In terms of both PCS and MCS, those who did not work at all (unemployed) exhibited lower mean scores. This is consistent with most studies looking at the relationship between wealth and health (e.g., [60,61,62]). Those who had jobs mostly as support staff reported better HRQoL than daily wagers who held tenuous positions with heavy workloads. These findings resonate with a previous study that also reported that higher workloads are significantly associated with lower HRQoL [63]. Moreover, previous findings have also shown that heavy workloads are correlated with a variety of negative health outcomes including stress-related conditions, employee well-being, and physical effects which may contribute to lower HRQoL [60,61,62].

The mean PCS scores were significantly lower among female participants compared to among males (54.4 ± 22.63 vs. 60.95 ± 22.37), which is supported by other international studies conducted among migrants and/or slum dwellers in China, Germany, Iran, Italy, Kenya, and Thailand [18,42,64,65,66,67]. This may be due to any number of underlying factors including lack of access to health care, a lower social priority for women’s health, less decision-making opportunities, and lower income [68,69]. Lower MCS scores were found in participants who reported work-related stress in their current residence. The slum dwellers are on average uneducated and working in the informal sector which is often riskier and more uncertain, which might increase stress [70]. A study in the US found immigrants tend to engage in higher risk jobs than US-born workers due to low educational attainment and the language barrier [51]. Migrant workers are also often engaged in “3D” jobs (dirty, dangerous, and demeaning/degrading) and are also highly prone to occupational injuries [71,72].

Being divorced/widowed, having no education, longer duration of migration, and not working/ less working (≤8 h/day) were all significantly (*p* < 0.05) associated with lower PCS scores in bivariate analysis. Being female, being divorced/ widowed, longer duration of migration, and having no education were significantly (*p* < 0.05) associated with lower MCS scores in bivariate analysis. None of these factors were significant in the multiple regression analysis.

In summary, specific socio-demographic variables, migration-related stressors, and work-related risks were associated with the HRQoL of migrants dwelling in slum areas. As the majority of urban residents (47.2%) live in different slum areas in Bangladesh, the immediate attention should be taken to improve their HRQoL with urban-friendly health policies and services to ensure improvement in overall urban health [6]. Moreover, proper housing facilities, work safety, and health care services are needed in this respect to maintain the well-being of this group, who make significant contributions to the national economy.

### 4.1. Limitations

The study has several limitations that should be acknowledged when interpreting the results. Firstly, the study was cross-sectional and did not permit any cause and effect relationship to be inferred. A prospective study would be helpful to determine sequential relationships. Furthermore, although the participants were recruited using random sampling, the sample is unlikely to be representative of the migrants to urban slums because the slums under the surveillance in the UHDSS were not randomly selected. Despite the fact that the SF-12 instrument has been validated with other populations in Bangladesh, it has not been separately validated in slum dwellers. In addition, following many studies conducted in low- and middle-income countries (LMICs), we used the SF-12 cutoff score as 50 as per the standard for the American general population, since no specialized scores for the developing countries were found [22,37,40,47,73,74].

### 4.2. Recommendations

The findings suggest future research is needed in various urban settings including small towns and municipalities to obtain consolidated findings and compare health indicators across urban slum areas. The findings from this study can be enhanced by conducting in-depth qualitative investigations to elicit context-sensitive information to answer why and how the occupational issues or poor housing affecting the HRQoL of the urban poor. A more comprehensive study with prospective methods should be considered as part of any plan to improve the HRQoL among migrants dwelling in urban slum areas. To note, there is no standard SF-12 cutoff for urban poor people in LMICs. Thus, research about the psychometric characteristics along with cutoff point justification of the SF-12 among urban slum dwellers is also needed. This research would add to the body of knowledge by identifying important factors that contribute to the quality of life and well-being of low-income urban migrants. It is evident that migration stressors and occupational health hazards are the important determinants of health among these populations. Therefore, policy attention should be given to appropriately address these issues to improve the quality of life of the urban poor. While creating livelihood opportunities in rural areas is critical, the Government of Bangladesh needs to formulate and implement policies and programs to ensure access to quality and essential health services, improving housing and living conditions in urban slums. Consideration should also be given to occupational health and safety measures to reduce health risks in factories and other workplaces, including strengthening relevant laws and regulations to reduce health risks at the workplace [75].

## 5. Conclusions

The present findings provide baseline information about the HRQoL of a sample of urban slum dwellers who migrated from different parts of Bangladesh. The poor HRQoL was observed among migrant slum dwellers, especially among elderly people, females, and migrated for longer duration. The findings suggest the need for evidence-based strategies to direct government policies in order to improve the HRQoL of low-income migrant populations. Available urban social protection programs should include a comprehensive social safety net for the improvement of the slum infrastructure as well as proper health care and risk mitigation plans at workplaces.

## Figures and Tables

**Table 1:** Distribution of all categorical variables (*n* = 935).

Variables	*n* (%)
Age (Mean ± SD)	44.8 ± 12.7
Sex	
Male	428 (45.8)
Female	507 (54.2)
Marital status	
Married	839 (89.7)
Divorced/widowed	65 (7.0)
Separated	17 (1.8)
Never married	14 (1.5)
Education level	
No education	477 (51.0)
Primary school	295 (31.6)
Secondary school	146 (15.6)
Higher secondary school	17 (1.8)
Duration of migration	
1–5 years	104 (11.1)
6–10 years	154 (16.5)
11–15 years	122 (13.0)
15–20 years	164 (17.5)
>20 years	391 (41.8)
Source of income	
Job	127 (13.6)
Self-employed	158 (16.9)
Daily wager	575 (61.5)
Unemployed	57 (6.1)
Other	18 (1.9)
Reasons for migration	
For new job opportunity	395 (42.2)
For education	4 (0.4)
For family	466 (49.8)
For eviction	4 (0.4)
Other reasons	66 (7.1)
Working hours	
Not working	377 (40.3)
≤8 h	287 (30.7)
>8 h	271 (29.0)
Comparing housing environment (current vs. rural)	
Worse	508 (54.3)
Same as before	79 (8.4)
Better	348 (37.2)
Comparing toilet (current vs. rural)	
Worse	366 (39.1)
Same as before	84 (9.0)
Better	485 (51.9)
Comparing water supply (current vs. rural)	
Worse	542 (58.0)
Same as before	65 (7.0)
Better	328 (35.1)
Comparing housing space (current vs. rural)	
Worse	780 (83.4)
Same as before	41 (4.4)
Better	114 (12.2)
Work-related risk in the current residence	
High	803 (85.9)
Moderate	83 (8.9)
Not significant	49 (5.2)
Work-related stress in the current residence	
High	781 (83.5)
Moderate	73 (7.8)
Not significant	81 (8.7)

**Table 2:** Descriptive statistics and correlations among SF-12 subscales and its two dimensions.

Variables	Mean (SD)	1	2	3	4	5	6	7	8	9	10
1. PF	67.83 (32.22)	—	0.319 **^f^	0.422 **^f^	0.026 ^f^	0.266 **^f^	0.313 **^f^	0.282 **^f^	0.419 **^f^	0.673 **^f^	0.461 **^f^
2. RP	50.05 (46.88)	0.294 **^m^	—	0.470 **^f^	0.022 ^f^	0.287 **^f^	0.494 **^f^	0.269 **^f^	0.289 **^f^	0.793 **^f^	0.507 **^f^
3. BP	75.43 (30.65)	0.497 **^m^	0.422 **^m^	—	0.002 ^f^	0.260 **^f^	0.331 **^f^	0.226 **^f^	0.273 **^f^	0.736 **^f^	0.401 **^f^
4. GHP	36.28 (28.71)	0.026 ^m^	0.008 ^m^	−0.001 ^m^	—	−0.053 ^f^	−0.026 ^f^	−0.013 ^f^	0.013 ^f^	0.330 **^f^	−0.028 ^f^
5. SF	76.63 (29.53)	0.346 **^m^	0.362 **^m^	0.452 **^m^	−0.017 ^m^	—	0.256 **^f^	0.357 **^f^	0.382 **^f^	0.315 **^f^	0.675 **^f^
6. RE	55.67 (44.98)	0.173 **^m^	0.515 **^m^	0.273 **^m^	−0.012 ^m^	0.245 **^m^	—	0.279 **^f^	0.243 **^f^	0.470 **^f^	0.729 **^f^
7. GMH	56.36 (23.11)	0.244 **^m^	0.289 **^m^	0.311 **^m^	−0.123 *^m^	0.346 **^m^	0.217 **^m^	—	0.436 **^f^	0.312 **^f^	0.669 **^f^
8. VEF	54.42 (32.49)	0.428 **^m^	0.234 **^m^	0.276 **^m^	−0.034 ^m^	0.299 **^m^	0.186 **^m^	0.381 **^m^	—	0.395 **^f^	0.710 **^f^
9. PCS	57.40 (22.73)	0.681 **^m^	0.772 **^m^	0.726 **^m^	0.344 **^m^	0.456 **^m^	0.418 **^m^	0.300 **^m^	0.354 **^m^	—	0.552 **^f^
10. MCS	60.77 (22.51)	0.427 **^m^	0.548 **^m^	0.474 **^m^	−0.055 ^m^	0.663 **^m^	0.725 **^m^	0.617 **^m^	0.664 **^m^	0.577 **^m^	—

Note: SD, standard deviation; PF, physical functioning; RP, role limitation-physical; BP, body pain; GHP, general health perception; SF, social functioning; RE, role limitation-emotional; GMH, general mental health; VEF, vitality emotional functioning; PCS, physical component summary score = (PF + RP + BP + GHP)/4; and MCS, mental component summary score = (SF + RE + GMH + VEF)/4. ^m^ Correlations coefficient for male; ^f^ correlations coefficient for female. * *p* < 0.05; ** *p* < 0.01.

**Table 3:** Bivariate and multiple regression analyses by PCS score.

Variables	Mean (SD)	Unadjusted Estimates					Adjusted Estimates				
		B	SE	t	β	*p*-Value	B	SE	t	β	*p*-Value
**Age**	— —	−0.66	0.05	−12.14	−0.37	<0.001	−0.70	0.07	−9.55	−0.39	<0.001
**Sex**											
Male	60.95 (22.37)				Ref.					Ref.	
Female	54.4 (22.63)	−6.55	1.48	−4.43	−0.14	<0.001	−7.50	1.89	−3.96	−0.16	<0.001
**Marital status**											
Married	58.25 (22.47)	−10.06	6.05	−1.66	−0.13	0.097	5.77	5.84	0.99	0.08	0.324
Divorced/widowed	44.13 (21.42)	−24.17	6.61	−3.65	−0.17	<0.001	6.77	6.64	1.02	0.08	0.308
Separated	57.35 (28.92)	−10.95	8.10	−1.35	−0.06	0.177	8.04	7.77	1.03	0.05	0.301
Never married	68.3 (15.59)				Ref.					Ref.	
**Education level**											
No education	53.93 (23.11)	−14.08	5.55	−2.54	−0.31	0.011	−5.65	5.22	−1.08	−0.12	0.279
Primary school	60.11 (21.86)	−7.91	5.60	−1.41	−0.17	0.158	−5.40	5.24	−1.03	−0.11	0.302
Secondary school	62.03 (21.1)	−5.99	5.76	−1.04	−0.10	0.299	−6.07	5.35	−1.13	−0.10	0.257
Higher secondary school	68.01 (25.85)				Ref.					Ref.	
**Duration of migration**											
1–5 years	62.38 (20.14)				Ref.					Ref.	
6–10 years	62.54 (21.22)	0.16	2.84	0.06	<0.01	0.955	1.95	2.62	0.74	0.03	0.457
11–15 years	61.12 (20.45)	−1.26	2.98	−0.42	−0.02	0.672	2.13	2.77	0.77	0.03	0.443
15–20 years	58.77 (21.55)	−3.62	2.80	−1.29	−0.06	0.197	1.42	2.66	0.53	0.02	0.593
>20 years	52.32 (24.13)	−10.06	2.46	−4.08	−0.22	<0.001	1.82	2.54	0.72	0.04	0.473
**Source of income**											
Job	69.88 (19.66)				Ref.					Ref.	
Self-employed	59.1 (21.67)	−10.78	2.62	−4.12	−0.18	<0.001	−7.09	2.54	−2.79	−0.12	0.005
Daily wager	55.68 (22.34)	−14.20	2.15	−6.59	−0.30	<0.001	−8.43	2.21	−3.82	−0.18	<0.001
Unemployed	48.57 (22.78)	−21.31	3.50	−6.08	−0.22	<0.001	−10.37	3.95	−2.63	−0.11	0.009
Other	37.15 (25.5)	−32.73	5.53	−5.91	−0.20	<0.001	−17.37	5.55	−3.13	−0.11	0.002
**Reasons for migration**											
For new job opportunity	59.84 (21.94)	−12.03	11.37	−1.06	−0.26	0.290	−5.88	10.55	−0.56	−0.13	0.578
For education	71.88 (25.77)				Ref.					Ref.	
For family	55.74 (22.86)	−16.14	11.36	−1.42	−0.36	0.156	−10.08	10.51	−0.96	−0.22	0.338
For eviction	70.31 (42.81)	−1.56	16.00	−0.10	<−0.01	0.922	6.45	14.79	0.44	0.02	0.663
Other reasons	52.84 (23.59)	−19.03	11.65	−1.63	−0.21	0.103	−7.26	10.79	−0.67	−0.08	0.501
**Working hours**											
Not working	52.97 (22.86)	−10.50	1.78	−5.90	−0.23	<0.001	−0.87	2.28	−0.38	−0.02	0.702
≤8 h	57.49 (22.07)	−5.98	1.89	−3.16	−0.12	0.002	−2.76	1.82	−1.52	−0.06	0.130
>8 h	63.47 (21.91)				Ref.					Ref.	
**Work-related risk in the current residence**											
High	57.98 (22.96)	6.45	3.34	1.93	0.10	0.054	4.62	3.47	1.33	0.07	0.184
Moderate	55.27 (20.39)	3.74	4.09	0.91	0.05	0.361	2.70	4.54	0.60	0.03	0.552
Not significant	51.53 (22.08)				Ref.					Ref.	
**Work-related stress in the current residence**											
High	57.74 (22.81)	3.65	2.65	1.37	0.06	0.169	−2.59	2.78	−0.93	−0.04	0.352
Moderate	57.45 (20.54)	3.36	3.67	0.92	0.04	0.360	0.97	4.15	0.23	0.01	0.815
Not significant	54.09 (23.82)				Ref.					Ref.	

Note: SD, standard deviation; B, unstandardized regression coefficient; SE, standard error; β, standardized regression coefficient.

**Table 4:** Bivariate and multiple regression analyses by MCS score.

Variables	Mean (SD)	Unadjusted Estimates					Adjusted Estimates				
		B	SE	t	β	*p*-Value	B	SE	t	β	*p*-Value
**Age**	— —	−0.55	0.06	−10.00	−0.31	<0.001	−0.54	0.07	−7.33	−0.31	<0.001
**Sex**											
Male	63.29 (21.62)				Ref.					Ref.	
Female	58.65 (23.05)	−4.64	1.47	−3.15	−0.10	0.002	−2.29	1.92	−1.19	−0.05	0.234
**Marital status**											
Married	61.75 (21.96)	−10.39	5.98	−1.74	−0.14	0.083	2.05	5.93	0.34	0.03	0.730
Divorced/widowed	47.63 (24.58)	−24.51	6.54	−3.75	−0.28	<0.001	−0.80	6.75	−0.12	−0.01	0.905
Separated	53.24 (25.47)	−18.91	8.01	−2.36	−0.11	0.018	−3.28	7.89	−0.42	−0.02	0.678
Never married	72.14 (20.17)				Ref.					Ref.	
**Education level**											
No education	58.29 (23.52)	−13.04	5.52	−2.36	−0.29	0.018	−5.55	5.30	−1.05	−0.12	0.295
Primary school	62.34 (21.46)	−8.98	5.58	−1.61	−0.19	0.108	−6.02	5.32	−1.13	−0.12	0.258
Secondary school	64.47 (20.39)	−6.85	5.73	−1.20	−0.11	0.232	−5.55	5.44	−1.02	−0.09	0.308
Higher secondary school	71.32 (20.56)				Ref.					Ref.	
**Duration of migration**											
1–5 years	66.95 (19.46)				Ref.					Ref.	
6–10 years	65.23 (21.26)	−1.72	2.82	−0.61	−0.03	0.542	−0.25	2.66	−0.09	<−0.01	0.925
11–15 years	63.25 (22.16)	−3.70	2.96	−1.25	−0.06	0.212	−0.27	2.82	−0.10	<−0.01	0.924
15–20 years	61.28 (21.49)	−5.67	2.78	−2.04	−0.10	0.042	−1.30	2.70	−0.48	−0.02	0.631
>20 years	56.38 (23.49)	−10.56	2.45	−4.31	−0.23	<0.001	−0.16	2.58	−0.06	<−0.01	0.951
**Source of income**											
Job	70.95 (18.53)				Ref.					Ref.	
Self-employed	62.27 (21.73)	−8.68	2.61	−3.32	−0.14	0.001	−4.73	2.57	−1.84	−0.08	0.066
Daily wager	59.42 (22.57)	−11.54	2.15	−5.37	−0.25	<0.001	−4.85	2.24	−2.17	−0.10	0.031
Unemployed	55.07 (21.51)	−15.89	3.50	−4.55	−0.17	<0.001	−2.14	4.01	−0.53	−0.02	0.594
Other	37.08 (25.66)	−33.87	5.52	−6.14	−0.21	<0.001	−16.17	5.63	−2.87	−0.10	0.004
**Reasons for migration**											
For new job opportunity	63.13 (22.23)	−3.12	11.27	−0.28	−0.07	0.782	3.25	10.69	0.30	0.07	0.761
For education	66.25 (20.39)				Ref.					Ref.	
For family	59.58 (22.52)	−6.67	11.26	−0.59	−0.15	0.554	0.39	10.65	0.04	0.01	0.971
For eviction	44.69 (27.96)	−21.56	15.86	−1.36	−0.06	0.174	−13.23	14.99	−0.88	−0.04	0.378
Other reason	55.70 (22.76)	−10.55	11.55	−0.91	−0.12	0.361	1.42	10.94	0.13	0.02	0.897
**Working hours**											
Not working	56.03 (22.80)	−11.83	1.75	−6.75	−0.26	<0.001	−7.58	2.31	−3.29	−0.17	0.001
≤8 h	60.31 (22.05)	−7.55	1.86	−4.05	−0.15	<0.001	−5.86	1.84	−3.18	−0.12	0.002
>8 h	67.86 (20.81)				Ref.					Ref.	
**Work-related risk in the current residence**											
High	61.65 (22.79)	3.41	3.30	1.03	0.05	0.301	—	—	—	—	—
Moderate	53.72 (21.66)	−4.52	4.04	−1.12	−0.06	0.263	—	—	—	—	—
Not significant	58.24 (16.62)				Ref.						
**Work-related stress in the current residence**											
High	61.08 (22.47)	−1.93	2.62	−0.73	−0.03	0.463	−6.34	2.49	−2.55	−0.10	0.011
Moderate	54.95 (22.82)	−8.06	3.63	−2.22	−0.10	0.026	−10.25	3.40	−3.01	−0.12	0.003
Not significant	63.01 (22.12)				Ref.					Ref.	

Note: SD, standard deviations; B, unstandardized regression coefficient; SE, standard error; β, standardized regression coefficient.

## Data Availability

The datasets used and/or analyzed during the current study are available from the corresponding author on reasonable request.
